# RIFTT: Robust Identification of Fusion genes in Tumor Transcriptomes

**DOI:** 10.1186/s12859-026-06725-4

**Published:** 2026-10-30

**Authors:** Paul Kerbs, Lisa Latzko, Ulrich Mansmann, Kristian Unger, Philipp A. Greif, Sebastian Vosberg

**Affiliations:** 1 Department of Medicine III, LMU University Hospital, LMU Medizin, Ludwig- Maximilians-Universität München, Munich, Germany; 2 German Cancer Consortium (DKTK), partner site Munich, a partnership between DKFZ and LMU University Hospital Munich (LMU Klinikum), Munich, Germany; 3 Department of Medical Data Processing, Biometry and Epidemiology, LMU University Hospital, LMU Medizin, Ludwig-Maximilians-Universität München, Munich, Germany; 4 Department of Radiation Oncology, LMU University Hospital, LMU Medizin, Ludwig- Maximilians-Universität München, Munich, Germany; 5 Bavarian Center for Cancer Research (BZKF), Partner Site Munich, Munich, Germany

**Keywords:** Fusion gene detection, Transcriptomics, RNA-seq, Cancer genomics, Computational oncology

## Abstract

**Background:**

Fusion genes serve as crucial biomarkers for cancer diagnosis, prognosis, and therapy, yet many detection tools lead to high false-positive rates and poor consensus. We developed RIFTT, a pipeline for Robust Identification of Fusion genes in Tumor Transcriptomes which integrates Arriba and FusionCatcher, two state-of-the-art fusion callers, together with novel scoring metrics (Promiscuity Score, Fusion Transcript Score, and Robustness Score).

**Results:**

Benchmarking on TCGA-LAML, BeatAML, and LL-100 datasets demonstrated RIFTT’s ability to reduce the number of candidates by up to 95% compared to single callers. At the same time, it preserves high-confidence events, outperforming both the nf-core/rnafusion pipeline and an Arriba/FusionCatcher consensus approach. RIFTT also identified novel high-evidence candidates, including a previously unreported recurrent fusion in acute myeloid leukemia.

**Conclusion:**

Packaged in a Singularity container for ease of use across computing environments, RIFTT provides standardized visual and annotated tabular outputs to support downstream analysis in research workflows and clinical molecular diagnostics. RIFTT is freely available at https://github.com/pkerbs/RIFTT.

**Supplementary information:**

The online version contains supplementary material available at https://doi.org/10.1186/s12859-026-06725-4.

## Background

Fusion genes are considered to be major biomarkers in multiple human cancers and their identification is crucial for diagnosis, prognosis, treatment strategies, as well as monitoring of disease progression. Fusion events driving tumor diseases usually result from chromosomal rearrangements, such as translocations, inversions, duplications, or deletions which relocate gene fragments (or complete genes) to other loci, potentially resulting in the formation of chimeric gene sequences. Furthermore, also aberrant transcription and trans-splicing events may lead to the formation of fusion genes.

The advent of RNA sequencing (RNA-seq) enabled rapid and transcriptome-wide readouts of genetic sequences with single nucleotide resolution allowing for accurate assembly and quantification of RNA transcripts. Over the last decade, many algorithms have been developed for reliable identification of fusion genes in RNA-seq data such as Arriba [1], defuse [2], FusionCatcher [3], JAFFA [4], SOAPFuse [5], STAR-Fusion [6], or TopHat-Fusion [7]. Moreover, several comparative studies assessed the performance of these tools on simulated data as well as on real datasets and concluded that, overall, there is no prevailing method [6, 8, 9].

False positive calls in fusion transcript detection are common, which is reflected by high numbers of reported fusion events and low concordance between individual tools. In addition, false negatives can lead to missed clinically relevant fusions, impacting diagnosis and treatment decisions. The challenge of accurate identification of disease-relevant fusion transcripts can be addressed by complementary use of multiple detection methods and specific filtering strategies as suggested by prior studies [8, 9]. However, integrating various software packages into diverse computational environments often involves resolving numerous software dependencies, which can be complex and time-consuming. Moreover, combining multiple fusion callers, which are based on different strategies to filter for high evidence candidates, is not straight forward and likely results in missing true variants, in particular in the context of identifying novel, unreported fusions.

**Fig. 1:**
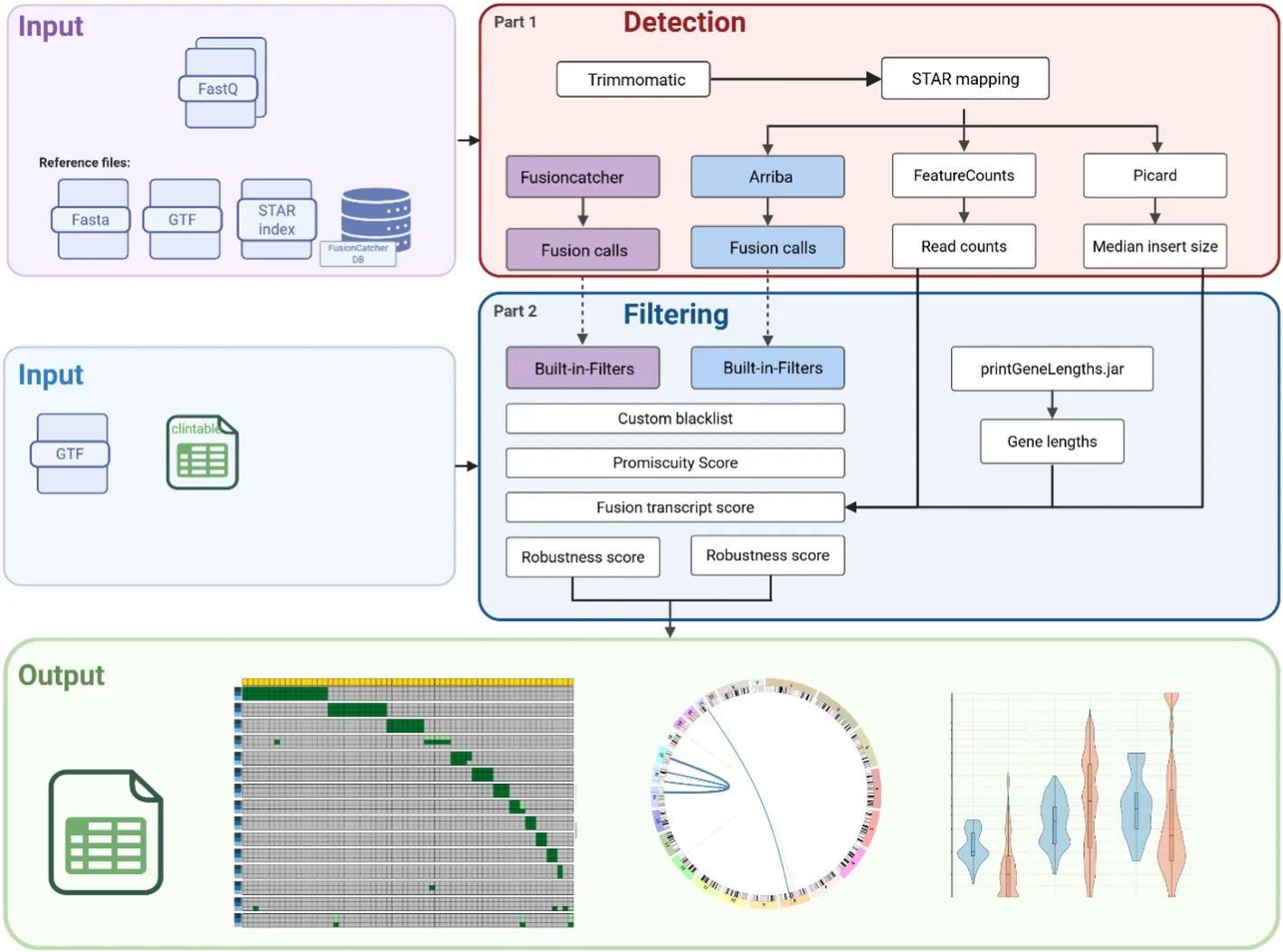
Overview of the RIFTT pipeline workflow. Part 1 identifies fusion genes based on two integrated fusion gene callers, Part 2 implements novel knowledge-based filtering metrics. The pipeline produces comprehensive outputs, including a tabular summary and visualizations of the result via an oncoprint, a circos plot, and violin plots for cutoff determination. Created in BioRender (https://BioRender.com/iujq6sm).

Therefore, we developed a pipeline for Robust Identification of Fusion genes in Tumor Transcriptomes (RIFTT) which incorporates fusion gene detection by two state-of-the-art fusion callers and knowledge-based filtering strategies (Fig. 1). All required modules and software dependencies were bundled into one application providing optimized compatibility with different system environments and simple usability for the identification of robust fusion gene candidates. RIFTT generates an oncoprint presenting a summary for high evidence known fusions and high evidence, recurrent unknown fusions, optionally incorporating clinical information, if provided (Supplementary Figure S1). RIFTT also generates a circos plot [10] for high evidence fusion events that were classified as robust calls (Supplementary Figure S2). Distribution of the PS and FTS values between known and unknown fusions presented in violin plots allow for the identification and adjustment of cutoffs (Supplementary Figure S3). Further, RIFTT reports evaluated and annotated fusion events as tabular output (Additional file 1). The table contains all relevant metrics which allow the user to perform individual post-filtering or re-evaluation of filtered events.

To benchmark our approach, we compared RIFTT against the underlying fusion detection algorithms, Arriba and FusionCatcher, against their overlapping candidate lists, as well as against another stand-alone approach, the widely used nf-core/rnafusion pipeline across *n* = 623 samples from three independent publicly available datasets. RIFTT reaches overall superior performance in terms of identifying originally reported, true fusions, while also providing high-evidence candidates for the discovery of novel fusion transcripts, however by yielding a substantially lower number of likely false-positive variants.

## Implementation

### Data

Fusion genes are well-established, clinically validated biomarkers in many tumor entities and play critical roles in disease classification, prognosis, and treatment decisions, especially in hematopoietic malignancies such as acute leukemias [11, 12], where fusion genes are detected in roughly 60% of cases, compared to below 10% in solid tumors [13, 14]. Detecting these fusions is a routine part of diagnosis and risk stratification, making it a highly relevant context to evaluate fusion detection methods.

In order to benchmark our RIFTT pipeline, we selected three representative hematologic datasets: TCGA-LAML [15] (*n* = 179), BeatAML [16] (*n* = 344) and the LL-100 panel [17, 18] (*n* = 100). Simulation of fusion data was not pursued, because performance on synthetic data does not reliably correlate with real-world tumor RNA-seq performance [9, 19]. To additionally assess generalizability beyond hematologic malignancies, we evaluated RIFTT on the TCGA-PRAD (Prostate Adenocarcinoma) dataset [20], a solid-tumor cohort with a previously published reference set of ETS family gene fusions (*n* = 333).

### RIFTT

RIFTT is divided into two major parts (Fig. 1). First, samples are processed by several open-source third-party tools to collect fusion calls, insert size, and read counts (Part 1). Secondly, data of all processed samples is collectively analyzed and annotated by specific filtering metrics to identify robust fusion gene candidates (Part 2). Both parts of this pipeline were bundled into a Singularity container [21] including all required software dependencies for easy portability between different system environments and user-friendly application.

#### Pre-processing and fusion calling (part 1)

Part 1 of the pipeline includes quality filtering of raw reads by fastP [22] and mapping by STAR [23]. The fusion calling step is performed using two independent fusion callers: Arriba [1], with trimmed and mapped reads, and FusionCatcher [3], on untrimmed and unmapped reads. Next, insert sizes are calculated by Picard toolkit (http://broadinstitute.github.io/picard/), and read counting is performed by Rsubread [24]. Expression is measured in TPM and normalization of fusion gene read counts is performed by substituting gene length with median insert size.

All output is organized in a structure that is required by Part 2 and stored in a specified folder. Detailed parameters and tool versions are enclosed in Supplementary Table S1.

#### Filtering (part 2)

Part 2 of the pipeline comprises seven filtering steps. In the first step, the built-in filtering metrics of the fusion callers are applied, and all reported fusion events are filtered by the number of supporting reads (minimum 3). Based on FusionCatcher reports, fusion events with an annotation (Supplementary Table S2) that implies irrelevant, non-somatic or false-positive events, as well as fusions whose partner genes showed sequence homology by common mapping reads are excluded. Based on Arriba reports, fusion events scored with a low confidence are excluded. In addition, fusion events that are listed on our custom generated blacklist (Additional file 2) are excluded. This blacklist contains fusion events that were previously called by Arriba and FusionCatcher in 49 publicly available healthy samples (Supplementary Table S3). Alternatively, a blacklist can be provided as optional input. All called fusions are evaluated based on external knowledge databases, ChimerDB [25] and MitelmanDB (https://mitelmandatabase.isb-cgc.org). For further filtering, we implemented three newly developed custom filter metrics, i.e., the Promiscuity Score (PS), the Fusion Transcript Score (FTS), and the Robustness Score (RS). Filtering of fusion genes based on these metrics requires defined cutoffs. As a knowledge-based approach, we classified called events into known and unknown fusion genes and evaluated both groups separately by our metrics to identify potential cutoffs. If recurrently present in ChimerDB (i.e., ≥ 2 PMIDs in ChimerKB/ChimerPub) and experimentally validated, by either PCR or Sanger, a fusion is defined as *known*.

Genes involved in fusions usually partner with one single other gene, and only a few genes are found to partner with many other genes [26]. Therefore, we defined the Promiscuity Score (PS) of a gene as the mean number of distinct fusion partners detected by the two incorporated fusion callers. The PS of a fusion is then calculated as the mean of the PS values of the two involved fusion partners. Since we observed unusually high PS values among unknown fusion events (Supplementary Figure S3a), those events are most likely artifacts and might be explained and therefore, we defined a PS-cutoff as the highest PS found among known fusion events.

Fusion gene expression is typically strongly correlated with the expression of its partner genes [26], which is the underlying assumption for our Fusion Transcript Score (FTS). Furthermore, most fusion artifacts arise from technical errors during sequence library preparation [27] as well as from sequencing errors and false mapping. Those artifacts are expected to have low or no correlation with expression of partner genes. To quantify this, for a fusion with 5′ partner gene A and 3′ partner gene B we define FTS5 = TPM_fusion / (TPM_fusion + TPM_A) and FTS3 = TPM_fusion / (TPM_fusion + TPM_B). TPM_fusion is derived from the breakpoint-spanning read count and TPM_A and TPM_B from read pairs covering the intact partner transcript adjacent to the breakpoint. All three counts are normalized to TPM using the Picard median insert size in place of gene length together with the sample’s transcriptome-wide scaling factor. FTS5 and FTS3 lie between 0 and 1 and can be interpreted as the allele frequency of the fusion relative to each partner gene. The Fusion Transcript Score of the fusion is FTS = (FTS5 + FTS3) / 2. We observed distinctively higher FTS values for known than for unknown fusion genes in our analyzed cohorts (Supplementary Figure S1b), with unknown fusions accumulating at low FTS. As a trade-off between sensitivity and specificity, a fusion passes the FTS filter if FTS5 ≥ 0.025, FTS3 ≥ 0.025, FTS5 < 1, FTS3 < 1 and FTS ≥ 0.05. The lower bound of 0.025 corresponds to a relative abundance of approximately 5% of the fusion-bearing clone in a bulk RNA-seq sample under the assumption of a monoallelic fusion; the upper bound of 1 excludes calls for which a partner gene shows no detectable wild-type transcription despite fusion-supporting reads, which is highly unlikely for a genuine fusion. The defined FTS cutoff separates fusion events from artifacts in most samples. In others those fusions were characterized by a FTS close to the cutoff and unusually high occurrence in a cohort. To filter these likely false positive events the Robustness Score (RS) was developed. The RS is defined as the ratio between numbers of samples within a cohort in which the FTS is passed to the total number of samples where the same fusion is called. Fusion genes were filtered if FTS criterion is not met in more than half of the samples (RS < 0.5).

Moreover, the pipeline identifies fusion genes that were consistently reported between the callers. All predicted fusions are then assigned to evidence levels based on the seven filtering steps (i.e., built-in filters, blacklist, PS, FTS, RS, database presence and caller overlap). We introduce evidence levels with a range from 0 (likely false event) to 7 (robust candidate), indicating how many filtering steps are passed.

Based on evaluations across our analyzed cohorts, we define an evidence level of 6 as indicating high-confidence candidates. This level was selected from Receiver Operating Curve (ROC) curve analysis, with a true positive rate of 0.76 and a false positive rate of 0.010 (Supplementary Table S4). Specifically, thresholds were chosen to balance sensitivity and specificity, ensuring that most true positives are retained while minimizing the contribution of false positives. Fusion calls meeting these criteria are considered robust.

### Evaluating performance of fusion gene detection

Results from RIFTT were compared both to the fusions originally reported (*OR*) in the corresponding publication [15–18] and to fusion calls detected by the nf-core/rnafusion pipeline (v3.0.2/Nextflow v24.10.4) [28, 29]. Both RIFTT and the nf-core/rnafusion pipeline were used with default settings. For the nf-core/rnafusion pipeline, ROC curve analysis identified an optimal score threshold of 0.375, which achieved a true positive rate of 0.65 at a false positive rate of 0.008. Increasing the cut-off showed an increase in additional candidates, while not retaining substantially more true positives (Supplementary Table S4). Similar to RIFTT, the nf-core pipeline applies a multi-caller approach in combination with reference knowledge databases. For a comparative ROC analysis across the three datasets, fusion genes described in the respective original publications [15–18] served as the reference set. However, establishing comprehensive ground truth for fusion genes is difficult, as orthogonal validations with for example FISH are practically limited to known disease-relevant fusions, while RNA-seq routinely uncovers novel candidates lacking targeted confirmation. For RIFTT, fusion calls with an evidence level of at least 6 are considered high evidence, while for the nf-core/rnafusion pipeline a cut-off of 0.375 is used, providing optimal performance given the ROC results (Supplementary Table S4).

Concordance of identified fusion genes was defined based on HGNC [30] gene symbols, i.e., a fusion was considered concordant if both partnering genes matched at the gene symbol level, regardless of the exact breakpoint position.

To assess the dependence of RIFTT’s cohort-relative scores (Promiscuity Score, Robustness Score) on cohort size and composition, each dataset was randomly down-sampled to *n* = 2, 5, 10, 20, 30, 50, 75, 100, 150, 200, 250, and 300 samples (restricted to sizes not exceeding the full cohort), with 100 independent random draws per size. For each sub-sample, Part 2 was re-run to recompute PS, maxPS, RS, and the resulting evidence level. For each call shared with the full-cohort analysis, we quantified the fraction whose high/low classification was unchanged and the fraction whose exact evidence level was unchanged.

For further evaluation of the individual contribution of each of RIFTT’s seven filtering criteria (caller built-in filters, blacklist, Promiscuity Score, Fusion Transcript Score, Robustness Score, caller overlap, and database evidence), we performed a leave-one-out ablation. Each criterion was removed individually and the evidence level recomputed. Performance was assessed using again OR fusions as ground truth, at a lowered operating point (evidence level ≥ 5, for FTS + RS ≥ 4).

## Results

### Detection of candidate fusions

**Fig. 2:**
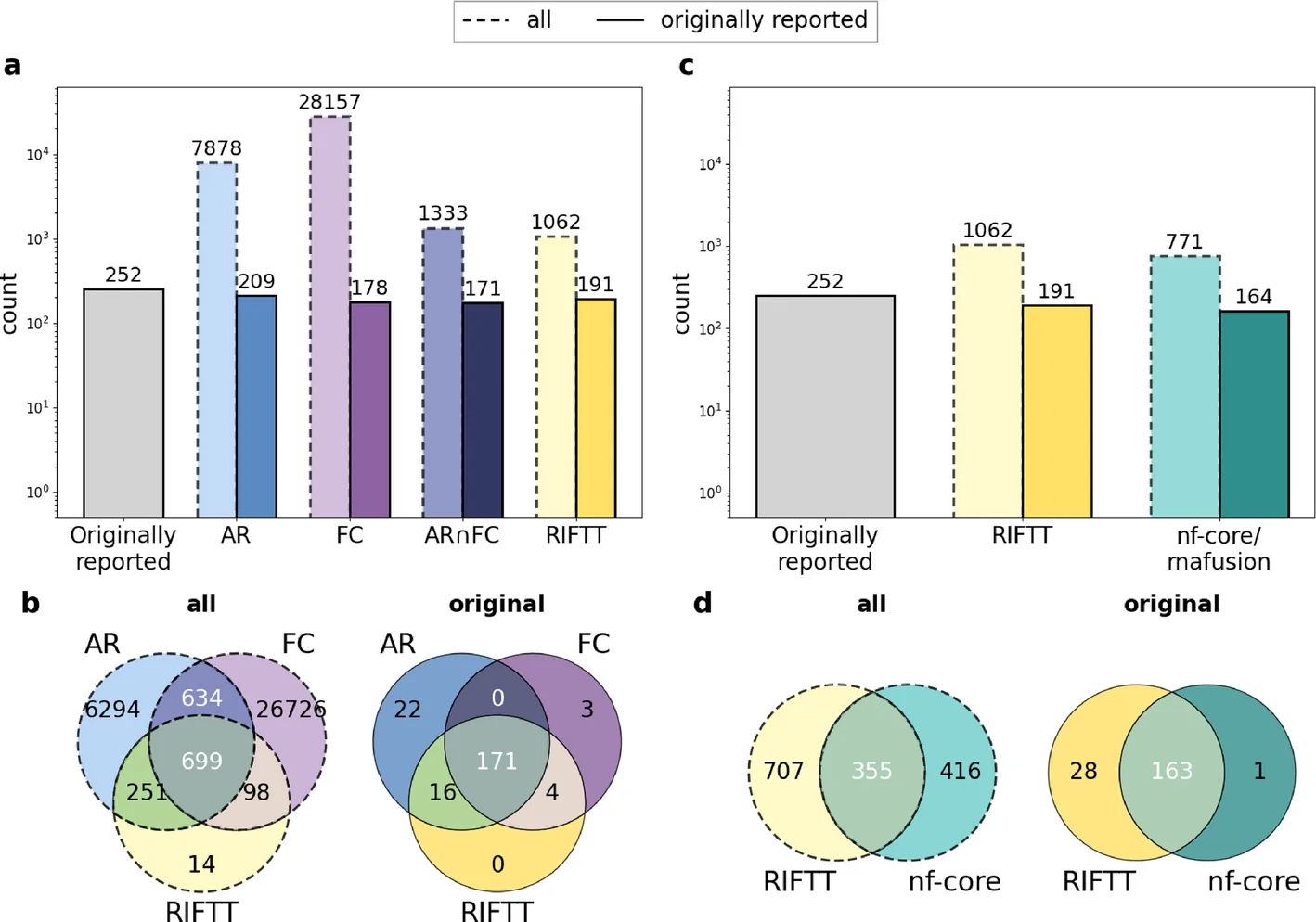
Concordance of fusions identified by RIFTT, its integrated fusion callers and the nf-core/rnafusion pipeline. **a** Numbers of fusion events originally reported (OR) across cohorts, and fusions called by Arriba (AR, blue), FusionCatcher (FC, purple), their overlap (dark purple) and RIFTT (yellow). **b** Overlap of fusions called by one or more approaches. **c** Numbers of fusion events originally reported (OR) across cohorts, fusions called by RIFTT (yellow) and nf-core/rnafusion (turquoise). **d** Overlap of fusion calls. Total numbers of fusion calls are shown in light colors and with dashed lines, overlaps with OR fusions are shown in dark colors and continuous lines

Across all three datasets (TCGA-LAML, BeatAML, LL-100) Arriba (AR) detected a total of 7,878 high evidence fusions and FusionCatcher (FC) identified 28,157 high evidence fusions after applying their respective built-in filters (Fig. 2a), however only a total of *n* = 1,333 fusions were overlapping (17% and 4.7% of all fusions identified with AR/FC, respectively, Fig. 2a, b). Using its knowledge-based filtering approach, RIFTT identified a total of 1,062 high evidence fusion candidates (Fig. 2a), including *n* = 699 supported by AR and FC (66%), *n* = 251 shared only with AR (24%) and *n* = 98 shared only with FC (9.2%, Fig. 2b). Additionally, *n* = 14 fusion genes were retained by RIFTT only, not supported by AR or FC (1.3%, Fig. 2b).

More specifically, in the TCGA-LAML dataset AR identified *n* = 1,817 fusions and FC reported *n* = 907 fusion genes after applying built-in filters (Supplementary Figure S4a), the overlap between AR and FC comprised *n* = 89 fusions (4.9% and 9.8%). RIFTT identified *n* = 104 high evidence fusion candidates, of which *n* = 83 fusions were also supported by AR and FC (80%). Additionally, RIFTT calls *n* = 19 fusions shared only with AR (18%) and *n* = 2 fusions shared only with FC (2%, Supplementary Figure S4b).

In the BeatAML dataset AR identified *n* = 3,362 fusions and FC reported *n* = 15,777 after built-in filtering (Supplementary Figure S4c), the overlap between AR and FC comprised *n* = 813 fusions (24% and 5.2%). RIFTT reported *n* = 361 high-evidence fusion candidates, of which *n* = 234 were also supported by AR and FC (65%). Additionally, RIFTT calls *n* = 58 fusions shared only with AR (16%) and *n* = 55 fusions shared only with FC (15%). Of note, RIFTT also called *n* = 14 fusion candidates that were missed by both, AR and FC (3.8%, Supplementary Figure S4d).

In the LL-100 dataset AR identified *n* = 2,699 fusions and FC reported *n* = 11,473 after built-in filtering, with *n* = 431 shared between both tools (16% and 3.8%, Supplementary Figure S4e). RIFTT reported *n* = 597 candidates, of which *n* = 382 were also supported by AR and FC (64%). Additionally, RIFTT calls *n* = 174 shared only with AR (29%) and *n* = 41 shared only with FC (6.9%, Supplementary Figure S4f).

### Detection of originally reported fusions by Arriba and FusionCatcher

Across all three datasets, a total of 252 fusion genes were described in the respective original publication [15–18], thus termed originally reported (OR) fusions here. AR identified *n* = 209 (83%) OR fusions, FC reported *n* = 178 (71%) OR fusions with an overlap between the two callers of *n* = 171 (68%) OR fusions. RIFTT retained *n* = 191 (76%) OR fusions (Fig. 2a). AR identified *n* = 22 OR fusions and FC identified *n* = 3 OR fusions not detected by RIFTT but also added *n* = 7,669 and *n* = 27,979 previously unreported fusion genes, respectively, while RIFTT added *n* = 871 previously unreported fusions (Fig. 2b).

In TCGA-LAML, *n* = 119 fusion genes were OR, with *CBFB*::*MYH11* (*n* = 22), *PML*::*RARA* (*n* = 16), and *RUNX1*::*RUNX1T1* (*n* = 7) being the most frequent. AR detected *n* = 97 (82%) OR fusions, FC detected *n* = 65 (55%) OR fusions. AR and FC overlap accounted for *n* = 65 (55%) OR fusions, while RIFTT retained *n* = 78 (66%) OR fusions (Supplementary Figure S4a). AR additionally identified *n* = 19 OR fusions not retained by RIFTT, while FC did not detect any additional OR fusion. However, they also added another *n* = 1,720 and *n* = 842 previously unreported fusions, respectively, while RIFTT added *n* = 26 unreported fusions, all shared with either AR or FC (Supplementary Figure S4b).

For the BeatAML dataset, only few clinically relevant fusions were described in the original literature, i.e., *CBFB*::*MYH11* (*n* = 22), *KMT2A*::*MLLT3* (*n* = 12), *PML*::*RARA* (*n* = 11), *RUNX1*::*RUNX1T1* (*n* = 8), *GATA2*::*MECOM* (*n* = 4), and *DEK*::*NUP214* (*n* = 3), a total of *n* = 60 OR fusions. AR detected *n* = 54 (90%) OR fusions, FC detected *n* = 55 (92%) OR fusions, AR and FC overlap accounted for *n* = 54 (90%) OR fusions, and RIFTT detected *n* = 55 (92%) OR fusions (Supplementary Figure S4c). Without reporting a single additional OR fusion in comparison to RIFTT, AR and FC reported *n* = 3,308 and *n* = 15,722 additional, not originally reported candidates, respectively (Supplementary Figure S4d). Instead, RIFTT reported one OR fusion missed by the overlap of AR and FC plus *n* = 306 additional candidates.

In the LL-100 dataset, *n* = 73 fusions were originally reported, with *BCR*::*ABL1* (*n* = 9), *IGH*::*MYC* (*n* = 8), *IGH*::*BCL2* (*n* = 7), and *IGH*::*CCND1* (*n* = 6) being the most frequent. AR detected *n* = 58 (79%) OR fusions, FC detected *n* = 58 (79%) OR fusions, AR and FC overlap accounted for *n* = 52 (71%) OR fusions, and RIFTT detected *n* = 58 (79%) OR fusions (Supplementary Figure S4e). A total of *n* = 3 OR fusions were called by AR but not by RIFTT, another *n* = 3 OR fusions were called by FC but not by RIFTT (Supplementary Figure S4f). However, AR had *n* = 2,641 and FC had *n* = 11,415 additional fusion calls, while RIFTT had *n* = 539. Moreover, RIFTT retained *n* = 6 OR fusions that were not included in the overlap of AR and FC.

### Detection of originally reported fusions by nf-core/rnafusion

Out of all *n* = 252 OR fusions, the nf-core/rnafusion pipeline identified *n* = 164 (65%) OR fusions of which *n* = 163 overlapped with the *n* = 191 (76%) OR fusion reported by RIFTT (Fig. 2c, d). In addition, the nf-core/rnafusion pipeline calls *n* = 607 additional, not originally reported fusions. Notably, both pipelines detected a substantial number of *SLC45A3*::*ELK4* fusions across all three cohorts, none of them being originally reported. The nf-core/rnafusion pipeline calls *n* = 61 of those fusions, RIFTT only calls *n* = 15 while *n* = 46 were classified as low evidence (Supplementary Figure S1).

Of the *n* = 119 OR fusions in the TCGA-LAML dataset, *n* = 75 (63%) were detected by the nf-core/rnafusion pipeline with high evidence, most of which (*n* = 74) overlapping with the *n* = 78 (66%) OR fusions detected by RIFTT (Supplementary Figure S5a). Three of the undetected OR fusions were recurrently reported within the cohort (*TFG*::*ADGRG7*, *n* = 3; *GAS6*::*TMEM255B*, *n* = 3; *NUP98*::*NSD1*, *n* = 3). In the case of *GAS6*::*TMEM255B* fusions, low evidence *GAS6-AS1*::*TMEM255B* fusions were detected by both pipelines in respective samples, in other cases these fusions were detected with low evidence (*NUP98*::*NSD1*, *n* = 1; *TFG*::*ADGRG7*, *n* = 3), by RIFTT and nf-core/rnafusion. Of the remaining *n* = 34 missed OR fusions, RIFTT detected *n* = 22 with low evidence. With respect to additional *n* = 9 OR fusion genes, RIFTT was able to call a fusion involving one of the reported partners, but not the second partner. Only three fusion genes were not detected at all by RIFTT (*BCL11A*::*GRIP2*, *CDK11B*::*SLC35E2A*, *FOXP1*::*PPP1R2*). In total, RIFTT detected *n* = 4 OR fusions that were not detected by the nf-core pipeline, while this pipeline detected *n* = 1 OR fusion not retained by RIFTT (Supplementary Figure S5b, Additional file 3).

Besides the OR fusions, RIFTT detected *n* = 26 additional fusions, while the nf-core pipeline detected *n* = 22 additional fusions (Supplementary Figure S5a). Notably, both pipelines identified an additional *BCR*::*ABL1* fusion with high evidence in the same sample that was not originally reported.

Of the *n* = 60 OR fusions in the BeatAML dataset, RIFTT and nf-core/rnafusion both detected *n* = 55 (92%) fusions with high evidence (Supplementary Figure S5c). Both approaches missed one *CBFB*::*MYH11* and all *n* = 4 described *GATA2*::*MECOM* fusion genes, none of them detected even with low evidence. RIFTT and nf-core/rnafusion both detected the same *n* = 55 OR fusions (Supplementary Figure S5d, Additional file 3).

Besides the OR fusions, RIFTT detected *n* = 306 additional fusions, while the nf-core pipeline detected *n* = 479 additional fusions with high evidence (Supplementary Figure S5c).

Of the *n* = 73 OR fusions in the LL-100 panel, RIFTT detected *n* = 58 (79%) fusions, while the nf-core/rnafusion pipeline detected only *n* = 34 (47%) with high evidence (Supplementary Figure S5e). Of the fusions missed by RIFTT, *n* = 11 (73%) involved *IGH* as a fusion partner, with seven of them being detected with low evidence, whereas all remaining *n* = 14 *IGH* fusions were reported with high evidence. In contrast, the nf-core pipeline did not detect any *IGH* fusion with high evidence, *n* = 25 *IGH* fusions were identified with lowest evidence only (score 0.062). For three of the fusion genes missed by RIFTT (*LMO1*::*TRD*, *n* = 1; *BCL11B*::*TLX3*, *n* = 2), at least one low evidence fusion event involving one partner could be detected in the respective sample. The nf-core/rnafusion pipeline detected the same fusions as RIFTT in those samples, *n* = 1 *LMO1*::*RIC3* fusion even with high evidence (score 0.375). In a single sample with a *BCL11B*::*NKX2.5* fusion, no evidence for this fusion was detected, neither with the nf-core/rnafusion pipeline. RIFTT detected *n* = 24 OR fusions that were not detected by the nf-core pipeline (Supplementary Figure S5f, Additional file 3).

Besides OR fusions, RIFTT detected *n* = 539 additional fusions, while the nf-core pipeline detected *n* = 106 additional fusions (Supplementary Figure S5e).

**Fig. 3:**
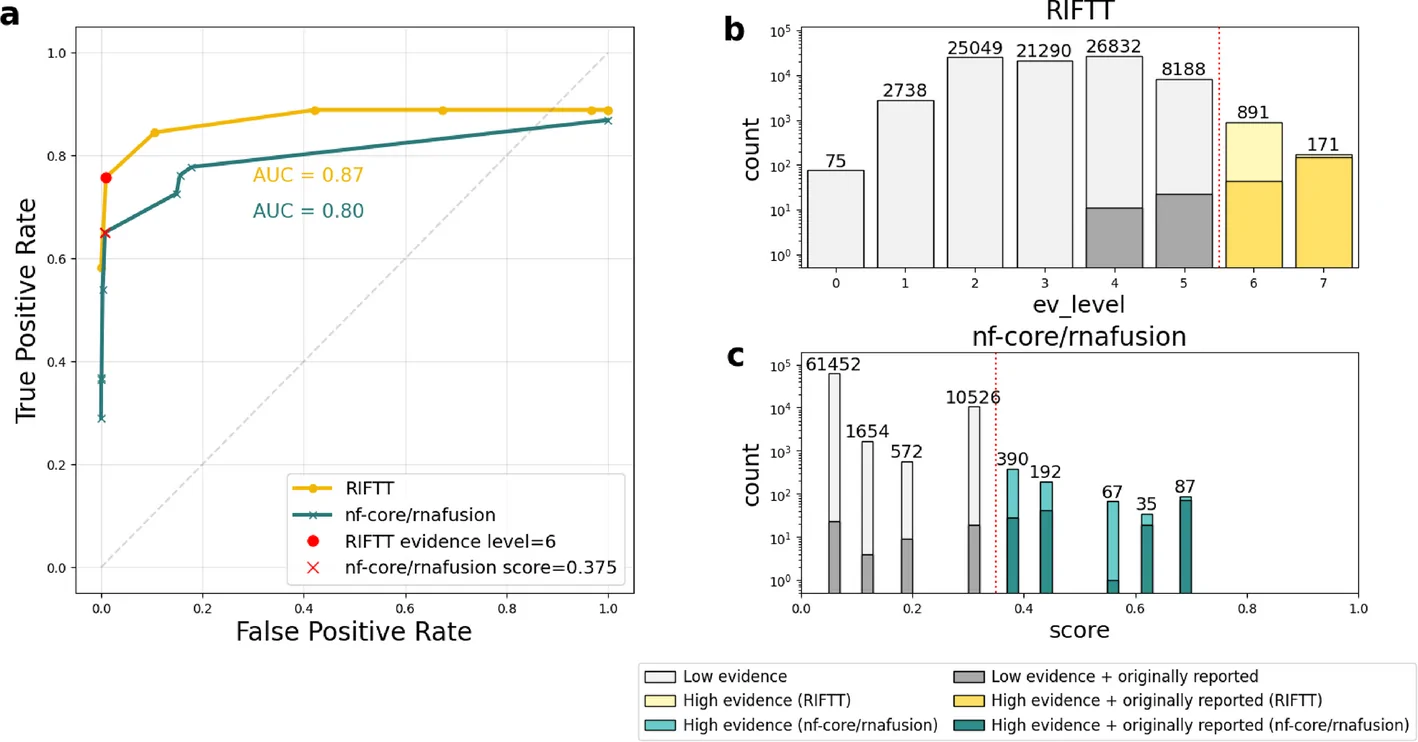
ROC analysis and distribution of evidence levels and scores. **a** Receiver Operating Characteristic (ROC) curves comparing RIFTT (yellow) and nf-core/rnafusion (turquoise) on all three datasets combined. True positive rate is plotted against false positive rate, based on originally reported genes as ground truth. Red marks indicate the determined cutoffs used for high evidence calling. AUC, Area Under the Curve. **b** RIFTT gene fusion evidence levels (ev_level) of all fusions identified. Grey colors indicate low evidence fusions, with all fusion calls in light grey and OR fusions in dark grey. Yellow colors indicate high evidence fusions, with all fusion calls in light yellow and OR fusions in dark yellow. High-evidence cutoff indicated in red. **c** nf-core/rnafusion scores of all fusions identified. Grey colors indicate low evidence fusions, with all fusion calls in light grey and OR fusions in dark grey. Turquoise colors indicate high evidence fusions, with all fusion calls in light turquoise and OR fusions in dark turquoise. High-evidence cutoff indicated by red dotted line

For a comparative ROC analysis across the three datasets, the OR fusion genes were considered as the ground truth. Across all three datasets RIFTT achieved an average AUC of 0.87 and the nf-core/rnafusion pipeline achieved an average AUC of 0.80. While the false positive rate was similar between the approaches (RIFTT: 0.010, nf-core/rnafusion: 0.008), RIFTT achieved a substantially higher true positive rate of 0.76, as compared to the nf-core/rnafusion pipeline which achieved a true positive rate of 0.65 (Fig. 3a). These values were based on the optimal cutoff for high evidence: an evidence level of minimum 6 in RIFTT and a score of minimum 0.375 in nf-core/rnafusion. A maximum AUC was achieved in the BeatAML dataset with 0.92 in both pipelines (Supplementary Figure S6).

### Distribution of confidence scoring

Evidence levels in RIFTT range from 0 (likely false event) to 7 (robust candidate). Examining the distribution of evidence levels across fusion calls, we observed that originally reported fusions consistently received high evidence scores, i.e., 4 to 7 within RIFTT (Fig. 3b). In contrast, the nf-core/rnafusion pipeline showed a broader distribution of evidence scores among OR fusions (Fig. 3c). In this pipeline, scores are given as values between 0 and 1, however in all three datasets no fusion call ever reached an evidence score close to the theoretical maximum score of 1. Instead, only the exact same nine different scores have been observed (i.e., 0.062, 0.125, 0.188, 0.312, 0.375, 0.438, 0.562, 0.625, 0.688) and OR fusions were described with scores across this entire range.

Notably, of the *n* = 78 OR fusions retained by RIFTT at high evidence in the TCGA-LAML dataset, the majority reached the highest evidence level 7 (*n* = 53/78, 68%, Supplementary Figure S7a), while *n* = 25 received an evidence level of 6. Of the remaining *n* = 41 OR fusions not retained at high evidence, *n* = 26 OR fusions received an evidence level of 5 or 4, and *n* = 15 fusions were not detected at all. Using the nf-core/rnafusion pipeline *n* = 37/75 (49%) OR fusions were called with high or very high scores (i.e., scores 0.562, 0.625, 0.688) while *n* = 38 OR fusions were called with scores close to the cutoff (i.e., score 0.375 or 0.438, Supplementary Figure S7b), *n* = 28 fusions were missed as they received scores below the high evidence cutoff and *n* = 16 were not detected at all.

In the BeatAML dataset, all OR fusions detected by RIFTT (*n* = 55) received the highest evidence level of 7 (Supplementary Figure S7c). While the nf-core/rnafusion pipeline called the same *n* = 55 OR fusions, only *n* = 31 received the highest observed score and *n* = 24 fusions received a score close to the high evidence cutoff (Supplementary Figure S7d). In both pipelines there was no evidence for the remaining *n* = 5 fusions.

In the LL-100 dataset *n* = 39/58 (67%) OR fusions called by RIFTT received an evidence level of 7, *n* = 19 OR fusions an evidence level of 6, *n* = 7 OR fusions an evidence level of 5 or 4, and *n* = 8 fusions remain undetected (Supplementary Figure S7e). In contrast, the nf-core/rnafusion pipeline called *n* = 25/34 OR fusions (74%) with the highest scores (0.625, 0.688) and *n* = 9 (26%) with a score close to the cutoff (0.375, 0.438, Supplementary Figure S7f). A total of *n* = 27 OR fusions were not called as they received a score below the cutoff, the majority (*n* = 17) even received the absolute lowest score observed (0.062), and *n* = 12 fusions were not detected at all.

### Discovery of a novel recurrent fusion gene using RIFTT

Moreover, the use of RIFTT has also facilitated novel discoveries. Independent of evidence levels and scores, RIFTT identifies more fusion candidates (*n* = 85,232) as compared to nf-core/rnafusion (*n* = 74,975), which can be also observed within all three cohorts: *n* = 9,814 vs. *n* = 7,000 in TCGA-LAML (+ 40%), *n* = 43,478 vs. *n* = 41,219 in BeatAML (+ 5%), and *n* = 31,942 vs. *n* = 26,756 in LL-100 (+ 19%) (Supplementary Figure S1). Importantly, RIFTT also retains more high-evidence candidate calls than nf-core/rnafusion (*n* = 1,062 vs. *n* = 771, + 38%). This indicates a greater potential to identify novel fusion transcripts by RIFTT. Within the fusion genes not listed in ChimerDB, *n* = 36 are recurrent fusion genes (TCGA-LAML *n* = 0, BeatAML *n* = 25, LL-100 *n* = 11, Supplementary Figure S2). Using RIFTT in our previous study [31] on a dataset of four cohorts, including also BeatAML, we identified *NRIP1*::*MIR99AHG* - a novel recurrent fusion gene that had remained unreported in those AML cohorts. The *NRIP1*::*MIR99AHG* fusion is the most recurring, not in ChimerDB listed fusion in the BeatAML cohort, when considering only fusions called with high evidence with AR and FC. Although the nf-core pipeline was also able to detect this fusion in the BeatAML dataset, it assigned it to such low confidence (score 0.188) that, under standard filtering thresholds, it would be excluded. For the nf-core/rnafusion pipeline to detect the *NRIP1*::*MIR99AHG* fusion, its confidence threshold would have had to be lowered to a score of 0.188, resulting in the output of more than 15 times as many candidate calls as with default settings (*n* = 8,008 vs. *n* = 534) and over 20 times more candidates than detected by RIFTT (*n* = 8,008 vs. *n* = 361), only in the BeatAML dataset.

### Stability of the scoring metrics across cohort size

The high/low classification agreed with the full-cohort result in ≥ 99.6% of shared calls already at *n* = 2, and the exact evidence level stabilized from *n* = 50 samples upward (Supplementary Figure S8a, b; Supplementary Table S5). The data-adaptive Promiscuity Score cutoff (maxPS) was strictly conservative under down-sampling, meaning a sub-cohort’s value was always less than or equal to the full-cohort value, reaching it only once the sub-sample covered approximately 85% or more of the cohort (Supplementary Figure S8c-e; Supplementary Table S6). Established driver fusions remained unaffected at every sampled cohort size down to *n* = 20.

### Contribution of individual filtering criteria

At the operating point, the full pipeline retained 1,062 high-evidence calls, recovering 191/252 (75.8%) originally reported fusions (AUC 0.871). Removing database evidence or caller overlap individually increased the candidate list 8- to 10-fold (to 9,170 and 8,936 calls, respectively). Database evidence also produced the largest single AUC loss (from 0.871 to 0.856). The caller built-in filter and the Fusion Transcript Score (together with the dependent Robustness Score) caused comparable AUC losses (− 0.013 and − 0.012, respectively) despite only modestly increasing the candidate list (+ 318 and + 596). Removing the Promiscuity Score, blacklist, or Robustness Score individually changed recovery by at most 2 fusions and AUC by ≤ 0.001 (Supplementary Figure S9a).

This pattern shifted per cohort. In TCGA-LAML, the caller built-in filter alone accounted for the largest AUC loss (− 0.032). In BeatAML, where baseline performance was already near-ceiling (AUC 0.916), all individual ablations changed AUC by ≤ 0.006. In LL-100, database evidence had an even larger effect than in the combined analysis (− 0.037), while removing the Fusion Transcript Score together with the Robustness Score improved AUC (+ 0.018). In contrast to TCGA-LAML, where the same ablation decreased AUC (− 0.015), and BeatAML, where it had no measurable effect (≤ 0.0005, Supplementary Figure S9b.

### Generalizability beyond hematologic malignancies

To assess whether RIFTT’s scoring framework generalizes beyond hematologic malignancies, we applied it to TCGA-PRAD. Of the 175 OR fusions, RIFTT detected 169 (97%) with high evidence and the remaining 6 with low evidence (Additional file 1). No originally reported fusion was missed entirely.

## Discussion

In this study, we show that RIFTT provides clear advantages over both individual fusion detection tools, Arriba and FusionCatcher, and the multi-caller nf-core/rnafusion pipeline. While Arriba and FusionCatcher identified thousands of candidate fusions with limited overlap, RIFTT’s knowledge-based filtering strategy consistently reduced noise, consolidating high-confidence calls that retained the majority of clinically and biologically relevant events. Across all three datasets, RIFTT preserved a larger fraction of originally reported fusions than Arriba and FusionCatcher, but with a dramatically reduced number of additional, likely spurious candidates. While the overlap of Arriba and FusionCatcher also reduces likely false positive calls, multiple originally reported fusions are missed by this approach, while retained by RIFTT. Importantly, RIFTT allows to rescue high-confidence fusions detected only by a single caller if these fusions meet the criteria of built-in filters while filtering out lower-quality events even when supported by multiple callers.

Importantly, RIFTT’s filtering strategy does not compromise discovery potential: RIFTT was able to identify a novel recurrent event, *NRIP1*::*MIR99AHG*, previously missed in AML cohorts, while it would have been excluded under standard settings using the nf-core/rnafusion pipeline, which was able to detect it at very low confidence only.

No single filtering criterion is responsible for RIFTT’s performance. Database evidence and caller overlap act as specificity anchors, removing either inflated the candidate list roughly 8- to 10-fold for a comparatively small gain in recovered fusions. The Promiscuity Score, blacklist, and Robustness Score each changed performance only marginally when removed individually. Not because they are redundant, but because a likely-false call typically fails several criteria simultaneously, so removing any single one is buffered by the others. This redundancy across complementary criteria, rather than the discriminative power of any one of them, is what makes RIFTT’s evidence scoring robust.

Evidence level thresholds proved to be important for interpreting results: levels 6 and 7 consistently represent robust candidates, in TCGA-LAML, all *n* = 53 calls with an evidence level of 7 were originally reported fusions, demonstrating RIFTT’s high specificity for the top evidence tier. Fusions with evidence levels 4 and 5 may still include true events, especially for fusions recurrently described in databases such as ChimerDB. Those fusion candidates require case-by-case evaluation within the context of the respective sample analyzed.

At the operating point, RIFTT recovers 76% of originally reported fusions while reducing the candidate list by up to 95% relative to the unfiltered call set. The 24% of originally reported fusions not retained by RIFTT fall into three groups. However, closer inspection revealed that these were either recurrently detected at low evidence levels (e.g. *NUP98*::*NSD1*, *TFG*::*ADGRG7*), reported with alternative partners not matching the exact originally reported annotation, or were likely reported based on a combined dataset of RNA-seq and karyotype data, suggesting that these fusions may not be detectable in RNA-seq alone, further supported by the fact that also the nf-core pipeline was unable to call these (*GATA2*::*MECOM* fusions in BeatAML). This illustrates that RIFTT’s conservative evidence thresholds effectively balance sensitivity with specificity, preventing inflation of candidate lists with low-confidence calls. Still, the tabular output retains all calls with their individual metrics, so lower-evidence candidates for a specific suspected fusion can be reviewed manually.

Compared with nf-core/rnafusion, RIFTT demonstrated superior sensitivity. Overall, it retained more originally reported fusions, achieved a higher AUC, and provided a more discriminative confidence scoring framework. In contrast to the coarse distribution of scores in nf-core/rnafusion, RIFTT assigned consistently high evidence levels to originally reported fusions, ensuring robust prioritization of clinically relevant events. This strength was particularly evident in the LL-100 dataset, where RIFTT retained originally reported fusions including immunoglobulin (*IGH*) partners, while nf-core/rnafusion failed to recover any *IGH* fusions with high evidence. In contrast to most tumor-specific fusion genes, which give rise to a fusion protein with distinct oncogenic properties, *IGH* rearrangements result in upregulation of their partner genes through juxtaposition of *IGH* locus enhancers. Given this fundamentally distinct underlying mechanism, *IGH* fusions are generally more difficult to detect by RNA sequencing data, also reported in a recent benchmarking study across 12 fusion-detection algorithms that identified IGH fusions among the most frequently overlooked driver events in hematologic malignancies [32]. However, RIFTT again demonstrates superior detection of *IGH* fusions compared to other approaches. Beyond the hematologic cohorts, RIFTT’s underlying scoring logic is not disease-specific. Consistent with this, RIFTT recovered 97% of originally reported fusions with high evidence in TCGA-PRAD, a solid-tumor cohort, suggesting the framework generalizes beyond blood cancers.

## Conclusion

Taken together, RIFTT unifies the strengths of multiple fusion callers while minimizing their individual weaknesses, delivering a superior balance of recall compared with both underlying tools and alternative multi-caller pipelines. Its robust evidence scoring system and ability to recover both well-established and novel recurrent events position RIFTT as a reliable and scalable framework for fusion transcript detection in both research and clinical contexts. This robustness arises from the deliberate redundancy across its seven filtering criteria rather than reliance on any single one, as shown by our ablation analysis. Looking ahead, RIFTT’s stringent yet sensitive approach offers strong potential for integration into diagnostic pipelines, where accurate fusion detection is critical for guiding therapy decisions. For example, detection of *BCR*::*ABL1*, *PML*::*RARA*, or other targetable fusions directly informs the use of tyrosine kinase inhibitors or differentiation therapy in leukemia. By improving confidence in the detection of such actionable events, while also supporting discovery of new oncogenic drivers, RIFTT provides a practical foundation for advancing precision oncology across diverse cancer types.

## Availability and requirements

Project name: RIFTT (Robust Identification of Fusion genes in Tumor Transcriptomes).

Project home page: https://github.com/pkerbs/RIFTT

Operating system(s): Linux (x86_64).

Programming language: R, Shell.

Other requirements: Singularity ≥ 3.6; ≥ 40 GB RAM; GENCODE genome reference (FASTA) and gene annotation (GTF); STAR index; FusionCatcher genomic database. 

Any restrictions to use by non-academics: None

## Supplementary information

Below is the link to the electronic supplementary material.

Additional file 1

Additional file 2

Additional file 3

Supplementary Material 4

## Data Availability

The RIFTT Singularity container, the source code and the documentation are available at (https:/github.com/pkerbs/RIFTT). TCGA and BeatAML RNA-seq data were accessed via the database of Genotypes and Phenotypes (dbGaP) under approved data access requests (dbGaP study accession: phs000178 (TCGA-LAML) and phs001657(BeatAML)). RNA-seq data from the LL-100 panel is publicly available from the European Nucleotide Archive (ENA) under accession number PRJEB30312.

## Abbreviations

FTS: Fusion Transcript Score
OR: originally reported
PS: Promiscuity Score
RIFTT: Robust Identification of Fusion genes in Tumor Transcriptomes
RNA-seq: RNA sequencing
ROC: Receiver Operating Characteristic
RS: Robustness Score
