# Supplementary material for: RIFTT: Robust Identification of Fusion genes in Tumor Transcriptomes
Source: BMC Bioinformatics. 2026 Oct 30;27:257. doi: 10.1186/s12859-026-06725-4 (PMC13729477; doi:10.1186/s12859-026-06725-4)
Supplement: Supplementary file 1 [file 12859_2026_6725_MOESM4_ESM.docx]

RIFTT: Robust Identification of Fusion genes
in Tumor Transcriptomes

Supplementary Information

Paul Kerbs^1,2*^, Lisa Latzko^1,2,*^, Ulrich Mansmann^3^, Kristian Unger^2,4,5^, Philipp A. Greif^1,2^, Sebastian Vosberg^1,2^

^1^ Department of Medicine III, LMU University Hospital, LMU Medizin, Ludwig-Maximilians-Universität München, Munich, Germany

^2^ German Cancer Consortium (DKTK), partner site Munich, a partnership between DKFZ and University

Hospital LMU Munich, Munich, Germany

^3^ Department of Medical Data Processing, Biometry and Epidemiology, LMU University Hospital, LMU Medizin, Ludwig-Maximilians-Universität München, Munich, Germany

^4^ Department of Radiation Oncology, LMU University Hospital, LMU Medizin, Ludwig-Maximilians-Universität München, Munich, Germany

^5^ Bavarian Center for Cancer Research (BZKF), Partner Site Munich, Munich, Germany

^*^ These authors contributed equally

Contact:

Corresponding author: Sebastian Vosberg ([sebastian.vosberg@med.uni-muenchen.de](mailto:sebastian.vosberg@med.uni-muenchen.de))

**Supplementary** **Figure 1**

**Figure S1. Comparison of detected fusion genes across cohorts.**

Fusion genes called by RIFTT, the nf-core/rnafusion pipeline, or originally reported across three cohorts: TCGA-LAML, BeatAML, and LL-100. Fusion genes that are recurrently called within a cohort and listed in ChimerDB are written in black. Fusions not listed in ChimerDB are shown, if recurrently reported in original literature (written in grey).

**Supplementary Figure 2**

**Figure S2. High evidence fusion genes.**

Fusion genes called by RIFTT with high evidence across three cohorts: **a** TCGA-LAML,
**b** BeatAML, and **c** LL-100.
Fusion genes listed in ChimerDB are connected by blue lines. Fusions not listed in ChimerDB are connected by grey lines, except if recurrently listed in the MitelmanDB (green lines), or recurrently reported within the cohort (red lines). Thickness represents recurrence.

**Supplementary Figure 3**

**a**

**b**

**Figure S3. Distribution of Fusion Transcript Score and Promiscuity** **Score.**
**a** Promiscuity Scores and **b** Fusion Transcript Scores for all fusion events across the datasets used: TCGA-LAML, BeatAML, and LL-100. Fusion events are stratified by their status as known (listed in ChimerDB, blue) or unknown (orange). Vertical dashed lines indicate cutoff determination for Promiscuity Scores.

**Supplementary Figure 4**

 **Figure S4. Concordance of fusions identified by RIFTT, and its integrated fusion callers.**

**a/b** Fusions called in TCGA-LAML. **a** Numbers of fusion events originally reported (OR) across cohorts, and fusions called by Arriba (AR, blue), FusionCatcher (FC, purple), their overlap (dark purple) and RIFTT (yellow). **b** Overlap of fusions called by one or more approaches. **c/d** Fusions called in BeatAML. **c** Numbers of fusions, **d** overlap of fusions. **e/f** Fusions called in LL-100. **e** Numbers of fusions, **f** overlap of fusions.
Total numbers of fusion calls are shown in light colors and with dashed lines, overlaps with OR fusions are shown in dark colors and continuous lines.

**Supplementary Figure 5**

**Figure S5. Concordance of fusions identified by RIFTT and nf-core/rnafusion.**

**a/b** Fusions called in TCGA-LAML. **a** Numbers of fusion events originally reported (OR) across cohorts, fusions called by RIFTT (yellow), and fusions called by nf-core/rnafusion (turquoise). **b** Overlap of fusion calls. **c/d** Fusions called in BeatAML. **c** Numbers of fusions, **d** overlap of fusions. **e/f** Fusions called in LL-100. **e** Numbers of fusions, **f** overlap of fusions.
Total numbers of fusion calls are shown in light colors and with dashed lines, overlaps with OR fusions are shown in dark colors and continuous lines.

**Supplementary Figure 6**

**Figure S6.** **Receiver Operating Characteristic (ROC) analysis**

True positive rate is plotted against false positive rate, based on originally reported genes as ground truth. Red marks indicate the determined thresholds used for high evidence calling. **a** ROC curves comparing RIFTT (yellow) and nf-core/rnafusion (turquoise) in TCGA-LAML. **b** ROC curves in BeatAML. **c** ROC curves in LL-100.
AUC, Area Under the Curve.

**Supplementary Figure 7**

 **Figure S7.** **Distribution of evidence levels and scores.**

**a/b** Fusions called in TCGA-LAML. **a** RIFTT gene fusion evidence levels (ev_level) of all fusions identified. Grey colors indicate low evidence fusions, with all fusion calls in light grey and OR fusions in dark grey. Yellow colors indicate high evidence fusions, with all fusion calls in light yellow and OR fusions in dark yellow. **b** nf-core/rnafusion scores of all fusions identified. Grey colors indicate low evidence fusions, with all fusion calls in light grey and OR fusions in dark grey. Teal colors indicate high evidence fusions, with all fusion calls in light teal and OR fusions in dark teal. **c/d** Fusions called in BeatAML. **c** RIFTT gene fusion evidence levels, **d** nf-core/rnafusion gene fusion scores. **e/f** Fusions called in LL-100. **e** RIFTT gene fusion evidence levels, **f** nf-core/rnafusion gene fusion scores.
High-evidence cutoff indicated by black dotted line.

**Supplementary Figure 8**

**Figure S8. Stability of evidence-level classification and Promiscuity Score cutoff across cohort size.**

Fraction of shared calls whose **a** exact evidence level and **b** high/low classification at evidence level ≥ 6 matched the full-cohort result, per cohort and sub-sample size; error bars show the 5^th^-95^th^ percentile across replicates. **c-e** Promiscuity Score cutoff (maxPS) per cohort. Points show the replicate mean, shaded bands the min/max and 5^th^-95^th^ percentile range, and the dotted line the full-cohort value.

**Supplementary Figure S9**

**Figure S9. Contribution of individual filtering criteria.**

Leave-one-out ablation of RIFTT's filtering criteria, shown separately per cohort. **b** TCGA-LAML. **c** BeatAML. **d** LL-100. Bars show the change in AUC (ΔAUC) when the indicated criterion is removed and the evidence level is recomputed at each ablation's adjusted operating point; labels give the corresponding change in recovered originally reported (OR) fusions and in the number of high-evidence candidate calls.  **Supplementary Table S1. Tool parameters and version**

| **FastP version 0.23.1** | **Rsubread version 2.10.4** | **STAR version 2.7.10a** |
| --- | --- | --- |
| -5 -3 -M 13 -W 1 -l 35 -p | featureCounts() function  Parameters: isPairedEnd = T ignoreDup = T useMetaFeatures = F allowMultiOverlap = T countChimericFragments = F | --outFilterMultimapNmax 50 --peOverlapNbasesMin 10 --alignSplicedMateMapLminOverLmate 0.5 --alignSJstitchMismatchNmax 5 -1 5 5 --chimSegmentMin 10 --chimOutType WithinBAM HardClip --chimJunctionOverhangMin 10 --chimScoreDropMax 30 --chimScoreJunctionNonGTAG 0 --chimScoreSeparation 1 --chimSegmentReadGapMax 3 --chimMultimapNmax 50 |
| **Arriba version 2.2.1** | **FusionCatcher version 1.33** | **Picard toolkit version 2.21.1** |
| -b <blacklist> (provided by Arriba) -k <known fusions> (provided by Arriba) -t <tags> (provided by Arriba) -p <protein domains> (provided by Arriba) | aligners=blat,star,bowtie,bowtie2  Required tools and versions: Bowtie version 1.2.3 Bowtie2 version 2.3.5.1 STAR version 2.7.10a Python version 2.7.18rc1 Biopython version 1.76 Blat version 0.35 SeqTK version 1.2-r101c Fastqtk version 0.27 BBMAP version 38.96 SAMTools version 1.14 Picard tools version 2.21.1 | CollectInsertSizeMetrics  M=0.5 |

**Supplementary Table S2. Discarded fusion events in FusionCatcher**

**Annotations of discarded fusion events as reported in column ‘Fusion_description’ by FusionCatcher**

1000genomes

banned

gap

gtex

partial

bodymap2

cacg

cortex

duplicates

ensembl_fully_overlapping

ensembl_partially_overlapping

ensembl_same_strand_overlapping

hpa

non_cancer_tissues

non_tumor_cells

pair_pseudo_genes

paralogs

readthrough

refseq_fully_overlapping

refseq_partially_overlapping

refseq_same_strand_overlapping

ucsc_fully_overlapping

ucsc_partially_overlapping

ucsc_same_strand_overlapping

pseudo

**Supplementary Table S3. Healthy samples.**

| **SRA Run ID** | **Tissue** | **Cell-Type** | **RNA selection** |
| --- | --- | --- | --- |
| SRR650429 | Bone Marrow | MNC | total RNA |
| SRR1575102 | Bone Marrow | CD34+ | poly(A) |
| SRR1575103 | Bone Marrow | CD34+ | poly(A) |
| SRR1575104 | Bone Marrow | CD34+ | poly(A) |
| SRR1575105 | Bone Marrow | CD34+ | poly(A) |
| SRR1660320 | Bone Marrow | CD34+ | total RNA |
| SRR1660321 | Bone Marrow | CD34+ | total RNA |
| SRR1660322 | Bone Marrow | CD34+ | total RNA |
| SRR1660323 | Bone Marrow | CD34+ | total RNA |
| SRR1660324 | Bone Marrow | CD34+ | total RNA |
| SRR5484559 | Bone Marrow | CD34+CD33+ | total RNA |
| SRR5952234 | Bone Marrow | CD34+ | total RNA |
| SRR5952235 | Bone Marrow | CD34+ | total RNA |
| SRR6781219 | Bone Marrow | CD34+ | total RNA |
| SRR6781220 | Bone Marrow | CD34+ | total RNA |
| SRR6781221 | Bone Marrow | CD34+ | total RNA |
| SRR6781222 | Bone Marrow | CD34+ | total RNA |
| SRR6781223 | Bone Marrow | CD34+ | total RNA |
| SRR6781224 | Bone Marrow | CD34+ | total RNA |
| SRR6781225 | Bone Marrow | CD34+ | total RNA |
| SRR6781226 | Bone Marrow | CD34+ | total RNA |
| SRR6781227 | Bone Marrow | CD34+ | total RNA |
| SRR6781228 | Bone Marrow | CD34+ | total RNA |
| SRR6781229 | Bone Marrow | CD34+ | total RNA |
| SRR6781230 | Bone Marrow | CD34+ | total RNA |
| SRR6781231 | Bone Marrow | CD34+ | total RNA |
| SRR6781232 | Bone Marrow | CD34+ | total RNA |
| SRR6781233 | Bone Marrow | CD34+ | total RNA |
| SRR6781234 | Bone Marrow | CD34+ | total RNA |
| SRR6781235 | Bone Marrow | CD34+ | total RNA |
| SRR6781236 | Bone Marrow | CD34+ | total RNA |
| SRR6781237 | Bone Marrow | CD34+ | total RNA |
| SRR6781238 | Bone Marrow | CD34+ | total RNA |
| SRR6781239 | Bone Marrow | CD34+ | total RNA |
| SRR6781240 | Bone Marrow | CD34+ | total RNA |
| SRR6781241 | Bone Marrow | CD34+ | total RNA |
| SRR13425455 | Blood | CD34+ | rRNA depleted |
| SRR13425456 | Blood | CD34+ | rRNA depleted |
| SRR13425457 | Blood | CD34+ | rRNA depleted |
| SRR13425458 | Blood | CD34+ | rRNA depleted |
| SRR13425459 | Blood | CD34+ | rRNA depleted |
| SRR13425460 | Blood | CD34+ | rRNA depleted |
| SRR13425461 | Blood | CD34+ | rRNA depleted |
| SRR13425462 | Blood | CD34+ | rRNA depleted |
| SRR13425469 | Blood | CD34+ | rRNA depleted |
| SRR13425480 | Blood | CD34+ | rRNA depleted |
| SRR17831321 | Bone Marrow | CD34+ | rRNA depleted |
| SRR17831323 | Bone Marrow | CD34+ | rRNA depleted |
| SRR17831324 | Bone Marrow | CD34+ | rRNA depleted |

**Supplementary Table S4. True and False positive rates in RIFTT and nf-core/rnafusion**

| **Score/Evidence Level** | **FPR** | **TPR** |
| --- | --- | --- |
| 0 | 1.000000 | 0.888889 |
| 1 | 0.999447 | 0.888889 |
| 2 | 0.967228 | 0.888889 |
| 3 | 0.672472 | 0.888889 |
| 4 | 0.421948 | 0.888889 |
| 5 | 0.106340 | 0.845238 |
| 6 | 0.010249 | 0.757937 |
| 7 | 0.000282 | 0.583333 |
| 0.062 | 0.999505 | 0.869048 |
| 0.125 | 0.178185 | 0.777778 |
| 0.188 | 0.156124 | 0.761905 |
| 0.312 | 0.148597 | 0.726190 |
| 0.375 | 0.008116 | 0.650794 |
| 0.438 | 0.003276 | 0.539683 |
| 0.562 | 0.001284 | 0.369048 |
| 0.625 | 0.000401 | 0.365079 |
| 0.688 | 0.000187 | 0.289683 |

**Supplementary Table S5. Evidence-level classification stability**

| cohort | metric | n | iter. | mean | min | max |
| --- | --- | --- | --- | --- | --- | --- |
| TCGA-LAML | high/low | 2 | 100 | 99.93 | 98.55 | 100.00 |
| TCGA-LAML | high/low | 5 | 100 | 99.94 | 99.16 | 100.00 |
| TCGA-LAML | high/low | 10 | 100 | 99.95 | 99.51 | 100.00 |
| TCGA-LAML | high/low | 20 | 100 | 99.98 | 99.79 | 100.00 |
| TCGA-LAML | high/low | 30 | 100 | 99.99 | 99.93 | 100.00 |
| TCGA-LAML | high/low | 50 | 100 | 99.997 | 99.96 | 100.00 |
| TCGA-LAML | high/low | 75 | 100 | 99.999 | 99.97 | 100.00 |
| TCGA-LAML | high/low | 100 | 100 | 99.999 | 99.98 | 100.00 |
| TCGA-LAML | high/low | 150 | 100 | 100.00 | 99.99 | 100.00 |
| TCGA-LAML | exact | 2 | 100 | 87.87 | 80.00 | 97.48 |
| TCGA-LAML | exact | 5 | 100 | 89.16 | 83.89 | 95.00 |
| TCGA-LAML | exact | 10 | 100 | 90.71 | 86.02 | 95.06 |
| TCGA-LAML | exact | 20 | 100 | 92.37 | 87.63 | 97.00 |
| TCGA-LAML | exact | 30 | 100 | 93.28 | 88.81 | 96.79 |
| TCGA-LAML | exact | 50 | 100 | 94.78 | 90.77 | 97.96 |
| TCGA-LAML | exact | 75 | 100 | 96.17 | 91.82 | 98.50 |
| TCGA-LAML | exact | 100 | 100 | 97.28 | 94.25 | 98.83 |
| TCGA-LAML | exact | 150 | 100 | 98.92 | 97.15 | 99.83 |
| BeatAML | high/low | 2 | 100 | 99.92 | 98.09 | 100.00 |
| BeatAML | high/low | 5 | 100 | 99.82 | 98.62 | 100.00 |
| BeatAML | high/low | 10 | 100 | 99.86 | 99.50 | 100.00 |
| BeatAML | high/low | 20 | 100 | 99.90 | 99.67 | 100.00 |
| BeatAML | high/low | 30 | 100 | 99.91 | 99.64 | 100.00 |
| BeatAML | high/low | 50 | 100 | 99.92 | 99.76 | 100.00 |
| BeatAML | high/low | 75 | 100 | 99.93 | 99.82 | 100.00 |
| BeatAML | high/low | 100 | 100 | 99.94 | 99.79 | 100.00 |
| BeatAML | high/low | 150 | 100 | 99.96 | 99.85 | 99.99 |
| BeatAML | high/low | 200 | 100 | 99.97 | 99.90 | 100.00 |
| BeatAML | high/low | 250 | 100 | 99.98 | 99.93 | 100.00 |
| BeatAML | high/low | 300 | 100 | 99.99 | 99.97 | 100.00 |
| BeatAML | exact | 2 | 100 | 82.80 | 69.04 | 91.12 |
| BeatAML | exact | 5 | 100 | 83.89 | 75.14 | 90.26 |
| BeatAML | exact | 10 | 100 | 85.43 | 76.14 | 91.78 |
| BeatAML | exact | 20 | 100 | 87.50 | 79.58 | 92.25 |
| BeatAML | exact | 30 | 100 | 88.66 | 82.11 | 93.43 |
| BeatAML | exact | 50 | 100 | 90.40 | 85.91 | 95.23 |
| BeatAML | exact | 75 | 100 | 91.48 | 86.50 | 94.98 |
| BeatAML | exact | 100 | 100 | 92.40 | 85.11 | 96.04 |
| BeatAML | exact | 150 | 100 | 94.62 | 88.77 | 97.49 |
| BeatAML | exact | 200 | 100 | 96.06 | 89.41 | 98.20 |
| BeatAML | exact | 250 | 100 | 97.48 | 90.97 | 98.74 |
| BeatAML | exact | 300 | 100 | 98.63 | 95.60 | 99.48 |
| LL-100 | high/low | 2 | 100 | 99.59 | 96.08 | 100.00 |
| LL-100 | high/low | 5 | 100 | 99.84 | 95.63 | 100.00 |
| LL-100 | high/low | 10 | 100 | 99.98 | 99.51 | 100.00 |
| LL-100 | high/low | 20 | 100 | 99.99 | 99.95 | 100.00 |
| LL-100 | high/low | 30 | 100 | 99.99 | 99.97 | 100.00 |
| LL-100 | high/low | 50 | 100 | 99.99 | 99.98 | 100.00 |
| LL-100 | high/low | 75 | 100 | 100.00 | 99.99 | 100.00 |
| LL-100 | exact | 2 | 100 | 88.05 | 53.17 | 96.14 |
| LL-100 | exact | 5 | 100 | 89.69 | 52.16 | 95.40 |
| LL-100 | exact | 10 | 100 | 92.48 | 83.17 | 95.79 |
| LL-100 | exact | 20 | 100 | 94.52 | 89.38 | 96.59 |
| LL-100 | exact | 30 | 100 | 95.75 | 92.98 | 97.10 |
| LL-100 | exact | 50 | 100 | 97.31 | 95.11 | 98.33 |
| LL-100 | exact | 75 | 100 | 98.68 | 97.65 | 99.11 |

**Supplementary Table S6. Promiscuity Score cutoff stability**

a) **TCGA-LAML**

| n | mean | min | max | p5 | p95 | sd |
| --- | --- | --- | --- | --- | --- | --- |
| 2 | 1.86 | 1.00 | 2.00 | 1.25 | 2.00 | 0.302 |
| 5 | 1.52 | 1.00 | 2.25 | 1.00 | 2.00 | 0.385 |
| 10 | 1.58 | 1.00 | 2.75 | 1.25 | 2.25 | 0.381 |
| 20 | 2.01 | 1.25 | 3.50 | 1.25 | 3.00 | 0.523 |
| 30 | 2.42 | 1.25 | 3.75 | 1.74 | 3.25 | 0.571 |
| 50 | 2.97 | 1.75 | 4.25 | 1.75 | 4.00 | 0.609 |
| 75 | 3.67 | 2.25 | 5.00 | 2.50 | 4.50 | 0.617 |
| 100 | 4.16 | 3.00 | 5.25 | 3.25 | 5.00 | 0.536 |
| 150 | 4.98 | 4.00 | 5.50 | 4.50 | 5.25 | 0.257 |

b) **BeatAML**

| n | mean | min | max | p5 | p95 | sd |
| --- | --- | --- | --- | --- | --- | --- |
| 2 | 1.86 | 0.75 | 2.25 | 1.00 | 2.00 | 0.343 |
| 5 | 1.51 | 0.75 | 2.25 | 1.00 | 2.00 | 0.412 |
| 10 | 1.74 | 1.00 | 4.50 | 1.25 | 2.50 | 0.543 |
| 20 | 2.40 | 1.50 | 5.25 | 1.74 | 3.26 | 0.596 |
| 30 | 3.05 | 1.75 | 6.00 | 2.24 | 4.50 | 0.731 |
| 50 | 4.23 | 2.75 | 8.00 | 3.00 | 5.76 | 0.962 |
| 75 | 5.52 | 3.25 | 10.50 | 4.00 | 7.52 | 1.181 |
| 100 | 6.77 | 4.25 | 11.25 | 4.99 | 9.26 | 1.472 |
| 150 | 9.53 | 6.00 | 14.50 | 6.99 | 12.26 | 1.847 |
| 200 | 12.11 | 7.50 | 16.00 | 8.50 | 15.50 | 2.183 |
| 250 | 14.91 | 9.25 | 18.50 | 10.24 | 17.76 | 2.132 |
| 300 | 17.71 | 12.00 | 20.00 | 15.50 | 19.51 | 1.519 |

c) **LL-100**

| n | mean | min | max | p5 | p95 | sd |
| --- | --- | --- | --- | --- | --- | --- |
| 2 | 1.99 | 1.00 | 4.50 | 1.00 | 3.26 | 0.653 |
| 5 | 3.52 | 1.25 | 11.50 | 1.50 | 6.79 | 1.985 |
| 10 | 6.26 | 1.50 | 15.50 | 3.21 | 12.30 | 2.716 |
| 20 | 11.55 | 6.50 | 21.00 | 7.00 | 19.25 | 3.633 |
| 30 | 16.01 | 8.50 | 24.75 | 10.50 | 23.29 | 4.104 |
| 50 | 23.59 | 13.50 | 31.25 | 17.25 | 29.50 | 4.029 |
| 75 | 32.70 | 25.75 | 37.75 | 26.75 | 36.26 | 2.760 |

**Additional material**

**Additional file 1**

.xlsx

RIFTT output

RIFTT output for TCGA-LAML, BeatAML and LL-100, TCGA-PRAD.

**Additional file 2**

.xlsx

Fusion blacklist

Blacklist generated from fusion calls in RNA-seq data of healthy samples, used for the exclusion of known artefacts and false-positive events.

**Additional file 3**

.xlsx

Originally reported fusions

Originally reported fusions and tools by which they have been detected.
